# Are Micro-Organisms Necessary to Pus Formation

**Published:** 1887-04

**Authors:** G. V. Black


ARTICLE II.

### ARE MICRO-ORGANISMS NECESSARY TO PUS FORMATION.

BY G. V. BLACK, M. D., D. D. S.

The thought of pathologists has been that the formation of pus is a vital act of the tissues and follows severe inflammation, or occurs in the healing of wounds, as one of the train of normal events. The idea that this process does not belong to the vital phenomena displayed by the tissues, has entered the minds of very few, perhaps; and with the observations of the past in which this event has so regularly followed in wounds or resulted from inflammations, it seems almost incredible that the time-honored opinion, one that has been accepted as a truism in all past ages, and down to the present, should now be questioned.

But very recently (*New York Medical Record*, December 25th, 1886), Dr. Knapp, an oculist of New York, has published views that are very significant, if not startling, on this subject, and he has summed up strict experimental evidence going far toward the establishment of the view that pus is not produced except in the presence of bacteria, and through their agency, that can no longer be ignored by pathologists.

There is no class of medical men to whom these matters come more closely in their daily practice than to dentists. We should certainly be abreast of the world in knowledge of these matters. Indeed we have to do directly with pus formation, and must take measures for its hindrance, or for its eradication, in every case in which we open the canals of a tooth's root; which, with those of us who are in full practice, is a thing of daily occurrence. This fact alone is sufficient to show that too much stress can not be laid upon gaining a correct understanding of this subject.

Dr. Knapp sums up the evidences that suppuration occurs only in the presence of micro-organisms under three questions:

"1st. Does traumatism of any kind produce suppuration?"

"2nd. Do foreign bodies occasion the formation of pus?"

"3rd. Are there any kinds of chemical agents that cause suppuration?"

On the first of these is cited the well known fact that subdermal fractures of the bones heal without suppuration. This fact is too well known to require any elaboration. But there are occasional exceptions to the rule, and these require some notice. The attention of clinicians has been strongly directed to this, and the search for the cause of this unusual suppuration has been vigorously followed. In this search it seems to have been pretty thoroughly established that in such cases there has been an established focus of suppuration in some other part of the body. And the suggestion that in such cases the pyogenic fungi have been transferred to the point of fracture from the abscess through the medium of the blood, has given rise to severe experimental tests with the view of ascertaining whether or not this is possible. Becker and Krause fractured the limbs of healthy animals and observed that they healed regularly without suppuration. They repeated these experiments in the same way, except that they injected pyogenic fungi into a vein of the ear (in rabbits) and found that in these cases the fractured limbs as regularly suppurated, and the same order of fungi as that injected was found in the pus, thus furnishing direct proof that pyogenic fungi may be conveyed to other parts by way of the circulation; and, while not necessarily producing injury at the point of inoculation, may produce suppuration in localities where *inflammatory exudates* give them a favorable nidus for development. This result has occurred with such regularity in these experiments that it is assumed by the experimenters that, in case suppuration occurs in subdermal fractures, this is a sufficient proof that a focus of suppuration exists somewhere in the body, whether such focus be found or not.

In addition to this testimony Dr. Knapp relates his own experiments in which he has lacerated, bruised, cut, and burned the eyes of rabbits in the most hideous way, aseptically, and uniformly without the production of pus; while comparatively delicate operations contaminated with pyogenic fungi as regularly suppurated. In many of his trials Dr. Knapp operated in the same way on each eye of the rabbit, one aseptically, the other contaminated with pyogenic fungi. The aseptic wound regularly healed by first intention, the septic as regularly suppurated. These operations were reported at length in the *Archives of Ophthalmology* last summer and the New York *Medical Record*, December 25th, 1886. As a criticism upon these two series of experiments taken altogether I would remark that it seems somewhat strange that fungi should not occasionally produce suppuration in the eye operated upon aseptically, having found their way thereto through the blood-streams, as in the experiments upon fractures.

The second question we do not need to discuss. I think it is fairly established that a foreign body does not, *per se*, produce suppuration. A bullet may lie in the flesh for years, shift its position by gravitation, etc., yet not cause the formation of pus. Other observations also go to establish the fact that foreign bodies do not of themselves produce suppuration. I may, however, mention the fact that Dr. Knapp thrust broken pieces of rusty hair-pins, previously brought to a glow, but not otherwise cleaned, into the eyes of rabbits and found that they did not cause suppuration. These were introduced with antiseptic precautions.

The third question is the most difficult of all, and the one upon which the greatest stress has been laid. Certainly if suppuration can not be produced by chemical irritants, the question of the production of pus by any form of injury *per se* must be given up, and the cause of this act must be sought without the tissues themselves. That is to say, the life forces resident within the tissues have not within the range of their unaided vital processes the power of the act of pus formation. This is the rather startling position to which we will be driven. I think such a proposition will be received by pathologists with great hesitation.

The proof of this proposition, as far as it is as yet developed, rests mainly upon the testimony of the experiments of five persons. These are J. Straus, E. Scheuerlen, Geo. Klemperer, J. A. Ruys and Dr. Knapp, all of whom seem to have performed their experiments in such a manner as to thoroughly test the possibilities in a practical way. I will not analyze all of the experiments but will simply indicate the plans of experimentation, and give the general results.

J. Straus used a sterilized tube tapering to a sealed point at one end, and closed with a sterilized plug at the other, containing a sterilized irritant. The skin of the animal was sterilized with the actual cautery, stabbed with a sterilized knife, the thin end of the tube introduced, broken within the tissues, and a few drops of croton oil, or other irritant, blown out into the tissues, the tube withdrawn, and the wound sealed with the actual cautery. Of eighteen injections of turpentine, five suppurated. Of five injections of croton oil, one suppurated. Of two injections of mercury, none suppurated. All of the cases of suppuration showed cocci in the pus, and were therefore considered as failures in obtaining perfect asepsis.

Scheuerlen's plans were better. Thin sterilized sealed tubes containing the sterilized irritant were placed under the skin with antiseptic precautions, and were allowed to remain ten days, or until the wound, through which they were introduced, had perfectly healed, and then they were broken, setting the irritant free in the tissues. A large number of experiments seem to have been made, using about a dozen irritants, among which were turpentine and croton oil. After breaking the tubes a hard swelling occurred at the spot, but no suppuration except in one case. In this one the experimenter concludes that the wound had not perfectly healed, and that cocci were in its track. Cocci were found in the pus.

Klemperer's experiments were made after the plans of Straus, and gave very similar results.

Drs. Ruys and Knapp injected croton oil and turpentine into the anterior chamber of the eyes of rabbits with antiseptic precautions. Here the effects could be seen, whitish deposits occurred which passed away after a time, but no suppuration in the larger number of cases, and in those in which pus was formed cocci were found also, showing that these had not been excluded.

The effect of these experiments certainly tends strongly to establish the proposition that without micro-organisms there is no such thing as pus formation. Farther than this the universal testimony of observers is that pus formation is confined mostly, if not entirely, to a specific form or family of micrococci, of which there are a number of species well known to mycologists. If I have read the results of experimentation aright, and observed aright (for I have also cultivated, and to a limited extent, experimented with these fungi,) this species of fungi is incapable of attacking normal living tissues. In case of subdermal fracture of a bone they produce suppuration at the point of fracture if injected into the veins of the ear, but do not produce suppuration at other points of the body. A focus of inflammation seems to create their opportunity. Many other organisms may attack living tissue without the aid of an inflammatory focus, but not these.

With these facts in view Dr. Knapp asks these questions and gives these answers. "What is pus? An albuminous non-coagulable fluid containing multitudes of leucocytes. What is suppuration? The splitting of living nitrogenous tissue into simpler compounds through the influence of certain bacteria." "In this way," says he, "the parallelism of the three processes—fermentation, putrefaction and suppuration—is established."

Now while the facts seem to drive us to some such conclusion as is intended to be formulated here, the formulation certainly can not remain as it is. I have no quarrel with the separation of fermentation and putrefaction, if it be understood that the distinction is simply between the nitrogenous and non-nitrogenous forms of one process which is in every case performed by some variety of fungi—bacteria, cocci, or bacilli. These facts need no demonstration now. But how can we conclude that pus formation is a variety of these processes? Certainly not through the idea of the disruption of the elements of living tissue. We have been taught that pus production consists of a certain form of albuminous exudate in which amœboid cells are found dead, and furthermore that many of the amœboid cells that come to the front in the healing of wounds die and float away in the purulent fluid, while a large proportion become built into a wall of living tissue, and as this is perfected the formation of pus is diminished. While there is a constant loss of living cells, the cells prevail in the end in all of the ordinary suppurations. I should therefore formulate the statement thus: *Suppuration results from the fermentative disruption of inflammatory exudates through the biological processes (or through the growth) of certain forms of cocci known as pyogenic fungi.* Or, as the cause of fermentation is so well understood in these days, the formulate might be limited thus: Pus formation is the result of a specific fermentation of inflammatory exudates. The word putrefaction might be substituted for fermentation as the material contains nitrogen, but from the fact that ill smelling products, characteristic of what we regard as putrefactive changes, are not present in the first instance. From my own observation I should conclude that the production of ill smelling products in pus is in every case due to a second process of disruption (this may go on contemporaneously), and by other germs than those that originally produce pus.

By the word inflammatory exudate here I intend to exclude the amœboid cells though recognizing them as being included in the exudate. No other part of the exudate can be regarded as living matter. The plastic portion is formed material that lies close to the very domain of life, but is not itself living. It is the proper matrix of the amœboid cells, which develop and go on to the formation of tissue within it. It disappears as the cells develop and form the living tissue, that finally closes the wound that has at first been sealed together by the plastic exudate. Now if this latter is disrupted, and rendered fluid, by a fermentative act, *i. e.*, a growth of pyogenic fungi, the amœboid cells die and become pus corpuscles. In this way we come to a formulation of the steps of the process of pus formulation that is at once rational, in harmony with the new facts obtained, and in accord with all of the teachings of science thus far developed, and yet without change in any of the material facts hitherto developed, except in the one idea that the formation of pus is a vital act of the tissues. According to this view, the production of pus is not a vital act of the tissue from whence it emanates, but an act of pyogenic fungi which take advantage of a definite form of opportunity.—*The Dental Review.*

